# Tobacco use and cessation strategies among individuals recently diagnosed with cancer

**DOI:** 10.1093/ntr/ntag145

**Published:** 2026-06-29

**Authors:** Amanda M Palmer, Kinsey Pebley, Andrew A Chen, Tracy T Smith, Alana M Rojewski

**Affiliations:** Department of Public Health Sciences, Medical University of South Carolina, Charleston, SC 29425, United States; Hollings Cancer Center, Medical University of South Carolina, Charleston, SC 29425, United States; Department of Public Health Sciences, Medical University of South Carolina, Charleston, SC 29425, United States; Hollings Cancer Center, Medical University of South Carolina, Charleston, SC 29425, United States; Department of Public Health Sciences, Medical University of South Carolina, Charleston, SC 29425, United States; Department of Public Health Sciences, Medical University of South Carolina, Charleston, SC 29425, United States; Department of Psychiatry and Behavioral Sciences, Medical University of South Carolina, Charleston, SC 29425, United States; Department of Public Health Sciences, Medical University of South Carolina, Charleston, SC 29425, United States; Hollings Cancer Center, Medical University of South Carolina, Charleston, SC 29425, United States

**Keywords:** cancer, cessation, electronic cigarettes, new tobacco products, special populations

## Abstract

**Introduction:**

Smoking cessation following a cancer diagnosis is critical for improving prognosis. For this reason, substantial efforts have been made to increase tobacco treatment in cancer care settings. The purpose of the present study is to provide an up-to-date estimate of tobacco/nicotine product use (inclusive of non-combustible products such as e-cigarettes and oral nicotine pouches) and cessation strategy uptake among individuals recently diagnosed with cancer.

**Methods:**

Data were derived from the Population Assessment of Tobacco and Health study, a large, nationally representative survey. Weighted frequencies estimated tobacco/nicotine product use (eg, cigarettes, cigars, e-cigarettes, oral pouches) and cessation strategy prevalence (eg, nicotine replacement therapies, prescription medications, e-cigarettes, oral pouches). Logistic regressions evaluated the associations between cancer diagnosis and cancer type (eg, tobacco related vs non-tobacco related cancers) on tobacco use and cessation strategy outcomes.

**Results:**

Individuals with cancer used tobacco/nicotine products at similar rates as those without cancer. Individuals with cancer were more likely to use evidence-based cessation strategies (eg, pharmacotherapy and counseling; Adjusted odds ratio [AOR] = 2.36 [95% CI, 1.30-4.27], *P* < .01), but appeared less likely to successfully quit (17.27% [5.42–29.13]) with cancer quit vs 31.16% [28.39–33.93] without cancer). Similarly, those with tobacco-related cancers appeared to have higher engagement with cessation strategies (11.90% [5.95–17.84]) than those with non-tobacco-related cancers (2.35% [0.74–3.95]).

**Conclusions:**

Efforts to increase reach and access to evidence-based cessation support in cancer settings have been fruitful; however, more work is needed to improve treatment effectiveness. Continued monitoring of the use of non-combustible products is warranted.

**Implications:**

Little is known about the contemporary prevalence of tobacco/nicotine product use and uptake of evidence-based and product substitution cessation strategies among individuals with cancer relative to those without cancer. Although they did not differ from non-cancer counterparts in terms of nicotine/tobacco use, individuals with cancer were more likely to use evidence-based cessation strategies, but appeared less likely to successfully quit. Non-cigarette product use was not a common smoking cessation strategy. Individuals with cancer are likely to seek evidence-based tobacco cessation support, but effectiveness could be improved.

## Introduction

Quitting smoking following cancer diagnosis has several benefits, including improved prognosis, decreased risk of complications and secondary disease, and significant healthcare cost savings.^1–3^ Yet, many patients who smoke face challenges in cessation, even after the teachable moment of diagnosis.^4^ For this reason, several efforts have been made to increase reach, access, and effectiveness of smoking cessation treatments within cancer care settings, such as the Cancer Center Cessation Initiative (C3I) funding initiative launched in 2017.^5^

First-line treatments for smoking cessation include behavioral support and pharmacotherapy, including nicotine replacement therapy, bupropion, and varenicline.^6^ However, over the past decade, the tobacco marketplace has shifted such that there is now increased availability of non-combustible products, such as e-cigarettes and oral nicotine pouches, both of which are sometimes considered by consumers as cessation aids.^7^^,^^8^ Data from 2013 to 2014 showed combustible cigarettes as the predominant tobacco product among lifetime cancer survivors (12.7%) followed by e-cigarettes (3.8%).^9^ During cessation attempts, these survivors endorsed using pharmacotherapy (34.1%), counseling (7.1%), and e-cigarettes (13.2%).^10^ Indeed, individuals with cancer appear to use more strategies than those without cancer in an effort to quit, with some strategies being evidence-based cessation strategies and others being non-evidence based.^11^ This may indicate a desire to quit among cancer survivors, but difficulty in achieving successful cessation. The present study provides an up-to-date estimate on combustible and non-combustible nicotine/tobacco product use and cessation strategies employed among those recently diagnosed with cancer.

## Methods

Data were from the Population Assessment of Tobacco and Health (PATH) study, which is a large, nationally representative, publicly available US survey of tobacco use and health outcomes.^12^^,^^13^ The Population Assessment of Tobacco and Health was approved by the Westat institutional review board. Data for the present analyses were derived from the Wave 7 (January 2022 to April 2023) adult cohort (18+; response rate, 52.4%).

Variables were self-reported (survey development has been previously published^13^; see Supplement for item details). Participants were asked if they had been told they had cancer by a medical professional in the past 12 months, and if so, to report the type of cancer which was further categorized into tobacco-related or not based on the Surgeon General’s report.^14^ Participants were also asked if they had used any of the following tobacco products, even just once, in the past 12 months: cigarettes, cigars (inclusive of traditional cigars, cigarillos, and little cigars), e-cigarettes, and oral nicotine pouches. Photos and descriptions of each product were included for clarity. Additional questions probed lifetime combustible product use (ie, former smoking). Participants endorsed whether or not they had attempted to or successfully quit combustible tobacco (cigarettes and/or cigars) in the past 12 months, and if so, they were asked to select any of the following strategies used during their most recent quit attempt: behavioral support (such as counseling, classes, or groups), mobile apps, nicotine replacement therapy, prescription medications (varenicline, bupropion), e-cigarettes, and oral nicotine pouches. Those who endorsed combustible tobacco use in the past 12 months and responded affirmatively to having successfully quit combustible tobacco were coded as abstinent at the time of the survey.

Analyses present past 12-month tobacco use and cessation strategy outcomes by cancer diagnosis (past 12 months [“survivors”] or none) and cancer type (tobacco-related or not). Fay’s balanced repeated replication weighting procedures were used for frequencies, and logistic regression models (without weighting), adjusted for demographics, estimated the association of cancer variables on outcomes. Tests were not performed for models with cell sizes <15. Weighted regression models were conducted, but results did not vary substantially and several models had convergence issues; therefore, these results are not presented but are included in Table SS2a and b. Analyses were conducted with SAS version 9.4 (SAS Institute).

## Results

The sample included *N* = 18 686 adults (43.71% [95% CI = 43.15% to 44.26%] male; 14.59% [14.08%–15.10%] Hispanic; 76.50% [75.90%–77.10%] White; see Table SS1). Use of tobacco/nicotine products did not differ between cancer (*N* = 361) and non-cancer groups (*N* = 18 325; Table 1). A higher proportion of cancer survivors using e-cigarettes (80.74% [64.19%–97.28%]) and nicotine pouches (60.15% [11.72–100.00]) were also smoking, compared to those without cancer (30.77% [28.70%–32.83%], 29.60% [24.34–34.86], respectively). Survivors were more likely to attempt smoking cessation (AOR = 1.50 [1.03–2.18], *P* < .05), but a lower proportion achieved abstinence (17.27% [5.42–29.13]) than those without cancer (31.16% [28.39–33.93]). Survivors were also more likely to use evidence-based cessation strategies (behavioral support, mobile apps, nicotine replacement therapy, and prescriptions) than those without cancer (AOR = 2.36 [1.30–4.27], *P* <.01).

**Table 1:** Tobacco use and quitting strategies by cancer status.

		No cancer diagnosis in past 12 months (*n* = 18 325)		Past 12-month cancer diagnosis (*n* = 361)			
		*n* ^a^	Weighted % (95% CI) ^b^	*n* ^a^	Weighted % (95% CI) ^b^	Adjusted OR (95% CI) ^c^	*P*
Overall ^d^	*N* = 18 686	18 325	97.08 (96.69-97.48)	361	2.92 (2.53-3.31)		
**Tobacco use**							
**Cigarette smoking**	**Past 12 months**	4765	16.68 (16.05-17.31)	117	15.78 (12.33-19.23)	1.071 (0.83-1.38)	.6016
**Cigar use** ^**e**^	**Past 12 months**	1986	6.89 (6.48-7.32)	38	5.76 (3.58-7.93)	1.35 (0.94-1.94)	.1091
**E-cigarette use**	**Past 12 months**	3415	9.66 (9.26-10.06)	32	4.98 (2.69-7.26)	1.18 (0.79-1.76)	.4152
	*Current combustible smoking*^f^	937	30.77 (28.70-32.83)	26	80.74 (64.19-97.28)	NA	NA
	*Former combustible smoking*^g^	1027	35.74 (33.78-37.70)	4	17.44 (0.91-33.98)	NA	NA
**Pouch use**	**Past 12 months**	439	1.35 (1.16-1.54)	7	1.19 (0.09-2.28)	NA	NA
	*Current combustible smoking*^f^	123	29.60 (24.34-34.86)	4	60.15 (11.72-100.00)	NA	NA
	*Former combustible smoking*^g^	152	42.62 (36.71-48.54)	1	9.69 (0.00-31.63)	NA	NA
**Quit attempts**							
**Combustible quit attempt** ^**h**^	**Past 12 months**	1805	31.76 (30.28-33.24)	62	41.99 (32.26-51.71)	1.50 (1.03-2.18)	.0347
	*Achieved abstinence*^i^	581	31.16 (28.39-33.93)	12	17.27 (5.42-29.13)	NA	NA
**Quit strategies** ^**^j^**^							
**Evidence-based strategy**	**Any evidence-based strategy**	481	28.10 (25.41-30.79)	33	55.20 (39.42-70.99)	2.36 (1.30-4.27)	.0048
	*Achieved abstinence*^k^	130	27.77 (22.98-32.57)	4	12.93 (0.00-27.08)	NA	NA
	**Behavioral support**	157	8.70 (7.06-10.34)	14	21.62 (12.73-30.52)	NA	NA
	*Achieved abstinence*^k^	45	25.53 (16.86-34.21)	2	15.15 (0.00-38.64)	NA	NA
	**Mobile app**	91	4.79 (3.57-6.00)	9	14.96 (5.55-24.36)	NA	NA
	*Achieved abstinence*^k^	31	32.49 (18.98-46.02)	2	21.91 (0.00-58.68)	NA	NA
	**NRT** ^l^	253	14.85 (12.78-16.93)	20	34.20 (18.07-50.32)	3.09 (0.35-27.47)	.3096
	*Achieved Abstinence*^k^	61	26.72 (20.59-32.85)	1	8.34% (0.00-25.45)	NA	NA
	**Any Rx** ^m^	115	19.87 (15.78-23.96)	8	31.34 (6.49-56.18)	NA	NA
	*Achieved abstinence*^k^	26	10.57 (6.04-15.10)	2	14.73 (0.00-35.89)	NA	NA
**Strategy: other product use**	**Any other product**	247	11.89 (10.28-13.50)	5	9.86 (0.00-19.92)	NA	NA
	*Achieved abstinence*^k^	81	31.77 (25.58-37.97)	0	NA	NA	NA
	**E-cigarette**	212	9.86 (8.41-11.31)	4	7.71 (0.00-16.67)	NA	NA
	*Achieved abstinence*^k^	72	32.13 (25.14-39.12)	0	NA	NA	NA
	**Pouches**	41	2.28 (1.40-3.15)	1	2.16 (0.00-6.68)	NA	NA
	*Achieved abstinence*^k^	10	14.89 (7.04-22.73)	0	NA	NA	NA

Due to instability of models, analyses with cell sizes less than 15 were not executed.

^a^Unweighted *n*. Ns and %s may not add up to full sample due to missing data.

^b^Weighted column %.

^c^Models included sex, age, race, ethnicity, education, and income, race, and ethnicity, and sex.

^d^Cancer diagnosis questions were only asked to respondents who endorsed seeing a doctor within the past 12 months (*N* = 18 686 adults of the full sample captured in the PATH study [*N* = 30 801]).

^e^Cigar products include traditional cigars, cigarillos, and filtered cigars.

^f^Current combustible smoking is defined as currently smoking cigarettes or cigar products every day or some days (and for combustible cigarettes only, >100 lifetime cigarettes). Weighted %s indicate the proportion of those endorsing the primary non-cigarette product who endorse current combustible tobacco use.

^g^Former combustible smoking is defined as having ever smoked cigarettes or cigar products fairly regularly (and for combustible cigarettes only, >100 lifetime cigarettes) but not within the past 12 months. Weighted %s indicate the proportion of those endorsing the primary non-cigarette product who endorse former combustible tobacco use.

^h^Participants were asked about strategies used during their most recent combustible tobacco quit attempt.

^i^Defined as those who endorsed past 12-month combustible tobacco use, but that they had quit at the time of the survey. Weighted %s indicate the proportion of column participants endorsing a quit attempt who had reported abstinence.

^j^Quit strategies were only asked to participants who had endorsed a quit attempt in the past 12 months. Participants could indicate multiple strategies.

^k^Weighted %s indicate the proportion of column participants who had endorsed this specific strategy and achieved abstinence.

^l^Nicotine replacement therapy.

^m^Chantix, varenicline, Wellbutrin, Zyban, and bupropion.

Abbreviation: NA, not available.

Survivors with tobacco-related cancers (*N* = 89) were more likely to be using cigarettes (AOR = 1.92 [1.03–3.58], *P* <.05) and e-cigarettes (AOR = 2.51 [1.04–6.10], *P* <.05) than survivors with non-tobacco-related cancers (*N* = 268; Table 2). Among survivors who reported e-cigarette use, most with (86.17% [64.16–100.00]) and without (73.77% [45.45–100.00]) tobacco-related cancers were also smoking. No significant difference in past-year quit attempts between tobacco- and non-tobacco-related cancers was found. A higher proportion of use of all quit strategies was observed for those with tobacco-related diagnoses relative to their counterparts.

**Table 2:** Tobacco use and quitting strategies of those diagnosed with cancer by cancer type.

		Non-tobacco-related		Tobacco-related			
		*n* ^a^	Weighted % (95% CI) ^b^	*n* ^a^	Weighted % (95% CI) ^b^	Adjusted OR (95% CI) ^c^	*P*
Overall ^d^	*N* = 361	268	80.26 (75.07-85.46)	89	19.74 (14.54-24.93)		
**Tobacco use**							
**Cigarette smoking**	**Past 12 months**	71	11.69 (8.21-15.17)	45	32.33 (19.37-45.28)	1.92 (1.03–3.58)	.0414
**Cigar** u**se**^**e**^	**Past 12 months**	26	5.57 (3.04-8.09)	12	6.87 (2.06-11.67)	NA	NA
**E-cigarette use**	**Past 12 months**	16	3.03 (1.06-4.99)	15	12.15 (2.80-21.50)	2.51 (1.04–6.10)	.0413
	*Current combustible smoking*^f^	12	73.77 (45.45-100.00)	13	86.17 (64.16-100.00)	NA	NA
	*Former combustible smoking*^g^	2	22.46 (0.00-51.13)	2	13.83 (0.00-35.84)	NA	NA
**Pouch use**	**Past 12 months**	3	0.78 (0.00-1.70)	4	2.93 (0.00-6.26)	NA	NA
	*Current combustible smoking*^f^	2	81.82 (36.95-100.00)	2	36.79 (0.00-92.13)	NA	NA
	*Former combustible smoking*^g^	0	NA	1	20.14 (0.00-61.81)	NA	NA
**Quit attempts**							
**Combustible quit attempt** ^**h**^	**Past 12 months**	31	29.15 (17.21-41.10)	30	59.63 (45.86-73.40)	2.02 (0.78-5.22)	.1457
	*Achieved abstinence*^i^	7	18.97 (3.10-34.84)	5	16.81 (0.00-34.42)	NA	NA
**Quit strategies** ^**j**^							
**Evidence-based strategy**	**Any evidence-based strategy**	15	2.35 (0.74-3.95)	17	11.90 (5.95-17.84)	2.25 (0.49–10.45)	.2901
	*Achieved abstinence*^k^	2	7.88 (0.00-19.60)	2	18.10 (0.00-40.88)	NA	NA
	**Behavioral support**	7	1.03 (0.07-1.99)	7	4.62 (1.08-8.16)	NA	NA
	*Achieved abstinence*^k^	1	8.20 (0.00-25.21)	1	21.45 (0.00-58.93)	NA	NA
	**Mobile app**	6	0.86 (0.00-1.74)	3	2.58 (0.00-5.21)	NA	NA
	*Achieved abstinence*^k^	1	9.76 (0.00-30.65)	1	38.47 (0.00-95.34)	NA	NA
	**NRT** ^l^	9	1.43 (0.24-2.62)	10	7.08 (1.52-12.64)	NA	NA
	*Achieved abstinence*^k^	0	NA	1	16.42 (0.00-47.97)	NA	NA
	**Any Rx** ^m^	8	1.22 (0.22-2.21)	4	1.36 (0.00-3.11)	NA	NA
	*Achieved abstinence*^k^	2	28.10 (0.00-60.90)	0	NA	NA	NA
**Strategy: Other product use**	**Any other product**	1	0.21 (0.00-0.61)	4	3.17 (0.00-7.30)	NA	NA
	*Achieved* a*bstinence*^k^	0	NA	0	NA	NA	NA
	**E-cigarette**	1	0.21 (0.00-0.61)	3	2.30 (0.00-5.85)	NA	NA
	*Achieved* a*bstinence*^k^	0	NA	0	NA	NA	NA
	**Pouches**	0	NA	1	0.88 (0.00-2.74)	NA	NA
	*Achieved* a*bstinence*^k^	0	NA	0	NA	NA	NA

Due to instability of models, analyses with cell sizes less than 15 were not executed.

^a^Unweighted *n*. Ns and %s may not add up to full sample due to missing data.

^b^Weighted column %.

^c^Models included sex, age, race, ethnicity, education, and income, race, and ethnicity, and sex.

^d^Cancer type was only asked to respondents who endorsed a cancer diagnosis within the past 12 months (*N* = 361). Tobacco-related cancers included bladder, cervix, colon, esophagus, kidney, larynx, liver, lung, mouth, pancreas, rectum, stomach, and throat. Non-tobacco related cancers included blood, bone, brain, breast, gallbladder, leukemia, lymphoma, melanoma, nervous system, ovarian, prostate, non-melanoma, unknown skin, soft tissue, testicular, thyroid, uterine, or other.

^e^Cigar products include traditional cigars, cigarillos, and filtered cigars.

^f^Current combustible smoking is defined as currently smoking cigarettes or cigar products every day or some days (and for combustible cigarettes only, >100 lifetime cigarettes). Weighted %s indicate the proportion of those endorsing the primary non-cigarette product who endorse current combustible tobacco use.

^g^Former combustible smoking is defined as having ever smoked cigarettes or cigar products fairly regularly (and for combustible cigarettes only, >100 lifetime cigarettes) but not within the past 12 months. Weighted %s indicate the proportion of those endorsing the primary non-cigarette product who endorse former combustible tobacco use.

^h^Participants were asked about strategies used during their most recent combustible tobacco quit attempt.

^i^Defined as those who endorsed past 12-month combustible tobacco use, but that they had quit at the time of the survey. Weighted %s indicate the proportion of column participants endorsing a quit attempt who had reported abstinence.

^j^Quit strategies were only asked to participants who had endorsed a quit attempt in the past 12 months. Participants could indicate multiple strategies.

^k^Weighted %s indicate the proportion of column participants who had endorsed this specific strategy and achieved abstinence.

^l^Nicotine replacement therapy.

^m^Chantix, varenicline, Wellbutrin, Zyban, and bupropion.

Abbreviation: NA, not available.

## Discussion

Results suggest that individuals with recent cancer diagnoses are using combustible products at similar rates as those without cancer. However, those with cancer who smoke may also engage in more concurrent use of non-combustible products (ie, e-cigarettes), although a small proportion did endorse e-cigarette use as a smoking cessation strategy. Compared to past reports,^10^ individuals with cancer show higher rates of quit attempts and use of evidence-based strategies (including counseling and pharmacotherapy). Despite this higher utilization, individuals with cancer are less likely to achieve abstinence than their counterparts. Those with tobacco-related cancers were observed to have higher proportions of tobacco/nicotine product use and higher use of cessation strategies. These results are consistent with prior analyses of Population Assessment of Tobacco and Health data suggesting that harm perceptions and health worry may uniquely impact cancer survivors and their cessation attempts.^15^

Recent efforts to expand tobacco treatment within cancer care (eg, the National Cancer Institute Cancer Center Cessation Initiative [C3I]) may have contributed to the observed increased uptake of evidence-based cessation strategies relative to the published 2013-2014 data.^10^ Indeed, evidence from C3I trials has demonstrated increased reach of tobacco treatment in cancer populations.^4^ However, the present results also echo evidence from C3I trials that underscore the need for additional work to increase treatment effectiveness.^16^ Historically, numerous trials have tested smoking cessation interventions tailored for cancer survivors.^17^ Although many past trials have been unsuccessful in yielding cessation rates higher than their control groups, some recent trials^18^^,^^19^ have shown promise. Thus, researchers are encouraged to continue to improve novel and innovative cessation resources to increase quit rates among this vulnerable population. Additionally, continued surveillance of non-combustible products among cancer survivors remains critical, as these products may be considered as appealing alternatives to traditional tobacco treatment, novel alternatives to cigarette smoking, or means by which to increase nicotine exposure in addition to smoking,^7^^,^^8^ as the present results suggest. Each of these motives for use can have important implications on cancer survivors’ health and cessation outcomes, especially as evidence on the health impacts of non-combustible products continues to accumulate. More data are needed to determine the effectiveness of non-combustible products for smoking cessation, their potential harm-reduction benefits, and potential risks to cancer survivors.

Limitations include low cell sizes that precluded some statistical comparisons and likely impacted the model stability. Additionally, the cross-sectional data lack temporality on when diagnoses and quit attempts were made in relation to one another, as well as inability to determine specificity of each strategy’s effectiveness for cessation outcomes. That is, some respondents may have attempted to quit smoking in response to a cancer diagnosis or after receiving cessation resources following their diagnosis. Alternatively, some respondents may have attempted to quit smoking prior to their diagnosis, perhaps in response to health symptoms or worry. Indeed, the selection and efficacy of each cessation strategy are likely influenced by the order of events. Finally, sociodemographics (eg, age differences between the samples with and without cancer) could have potentially contributed to outcomes uniquely or synergistically with cancer status. Overall, these data provide a foundation for future study of tobacco product use among individuals with cancer, which can inform the development of tailored treatments and improvement of cessation strategy effectiveness for this priority population.

## Supplementary Material

NTR_Supplement_RR_draft_2_-_clean_ntag145

## Data Availability

Data are publicly available at www.icpsr.umich.edu/web/NAHDAP/studies/36498/ to those who register with and agree to terms of use with ICPSR.
