# Supplementary material for: Tobacco use and cessation strategies among individuals recently diagnosed with cancer
Source: Nicotine Tob Res. 2026 Jun 29;28(11):1961–6. doi: 10.1093/ntr/ntag145 (PMC13725580; doi:10.1093/ntr/ntag145)
Supplement: Supplementary file 1 [file ntr_supplement_rr_draft_2_-_clean_ntag145.docx]

**Supplement**

to

Tobacco Use and Cessation Strategies Among Individuals Recently Diagnosed with Cancer

Amanda M. Palmer, PhD, Kinsey Pebley, PhD, Andrew A. Chen, PhD, Tracy T. Smith, PhD & Alana M. Rojewski, PhD

Contents:

1. Supplementary Table 1: Sample demographics
2. Supplementary Tables 2a and 2b: Weighted regression analyses results
3. Survey items

**Table 1.** Sample Demographics and Characteristics

|  |  | **No cancer diagnosis in past 12-months** | | **Past 12-month cancer diagnosis** | |  |
| --- | --- | --- | --- | --- | --- | --- |
|  |  | n^a^ | Weighted %  (95% CI)^b^ | n^a^ | Weighted %  (95% CI)^b^ | *Χ*^2^ (*p*) |
| **Overall** | N=18,686^c^ | 18325 | 97.08  (96.69 - 97.48) | 361 | 2.92  (2.53 - 3.31) | – |
| **Sex** | Male | 7499 | 43.71  (43.15 - 44.26) | 177 | 48.27  (42.81 - 53.74) | 4.48 (.11) |
|  | Female | 10802 | 56.29  (55.74 - 56.85) | 184 | 51.73  (46.26 - 57.19) |  |
| **Age group** | 18-24 | 5065 | 9.01  (8.84 - 9.18) | 12 | 0.62  (0.26 - 0.97) | 368.44 (<.0001) |
|  | 25-34 | 4360 | 15.36  (14.91 - 15.82) | 9 | 1.26  (0.35 - 2.17) |  |
|  | 35-44 | 2275 | 15.53  (14.99 - 16.06) | 25 | 5.54  (2.65 - 8.43) |  |
|  | 45-54 | 1938 | 15.80  (14.98 - 16.63) | 47 | 10.64  (6.47 - 14.80) |  |
|  | 55-64 | 2213 | 18.70  (17.88 - 19.52) | 94 | 24.52  (18.42 - 30.62) |  |
|  | 65 or older | 2473 | 25.59  (25.10 -26.08) | 174 | 57.43  (50.61 - 64.25) |  |
| **Race^d^** | Black alone | 2969 | 11.60  (11.26 - 11.95) | 56 | 8.07  (5.23 - 10.91) | 25.82 (<0.01) |
|  | White alone | 12800 | 76.50  (75.90 - 77.10) | 276 | 85.74  (81.05 - 90.44) |  |
|  | Other | 2019 | 11.90  (11.34 - 12.46) | 22 | 6.19  (2.06 - 10.31) |  |
| **Ethnicity** | Hispanic | 3456 | 14.59  (14.08 - 15.10) | 38 | 7.47  (1.99 - 11.42) | 21.58 (<.01) |
|  | Non-Hispanic white | 14589 | 85.41  (84.90 - 85.92) | 316 | 92.53  (88.58 - 96.49) |  |
| **Income** | <$50K | 8359 | 41.58  (40.46 - 42.70) | 176 | 44.29  (37.27 - 51.30) | 1.51 (.46) |
|  | $50K+ | 9028 | 58.42  (57.30 - 59.54) | 163 | 55.71  (48.69 - 62.73) |  |
| **Education** | Less than HS | 1377 | 7.55  (7.10 - 7.99) | 51 | 10.91  (6.34 - 15.47) | 13.85 (.19) |
|  | HS or GED | 4438 | 25.61  (24.97 - 26.24) | 82 | 24.60  (17.38 - 31.83) |  |
|  | Some college | 6731 | 30.14  (29.65 - 30.63) | 101 | 24.97  (19.18 - 30.76) |  |
|  | College grad | 5735 | 36.71  (36.07 - 37.34) | 125 | 39.52  (33.52 - 45.52) |  |

Notes:

^a^ Unweighted n. Ns and %s may not add up to full sample due to missing data.
^b^ Weighted column %.
^c^ Cancer diagnosis questions were only asked to respondents who endorsed seeing a doctor within the past 12 months (N=18,686 adults of the full sample captured in the PATH study [N=30,801])
^d^ The other category included Asian, multiple races, and other races.

**Table 2a.** Tobacco use and quitting strategies by cancer status: Results from weighted regression analyses

|  |  | **No cancer diagnosis in past 12-months (n=18,325)** | | **Past 12-month cancer diagnosis (n=361)** | |  | |
| --- | --- | --- | --- | --- | --- | --- | --- |
|  |  | **n^a^** | **Weighted %**  **(95% CI)^b^** | **n^a^** | **Weighted %**  **(95% CI)^b^** | **Adjusted OR (95% CI)^d^** | ***p*** |
| **Overall^c^** | N=18,686 | 18,325 | 97.08  (96.69 - 97.48) | 361 | 2.92  (2.53 - 3.31) |  |  |
| **Tobacco Use** | | | | | | | |
| **Cigarette Smoking** | **Past 12-month** | 4765 | 16.68  (16.05 - 17.31) | 117 | 15.78  (12.33 -19.23) | 1.20  (0.88 - 1.65) | 0.2529 |
| **Cigar Use^e^** | **Past 12-month** | 1986 | 6.89  (6.48 - 7.32) | 38 | 5.76  (3.58 - 7.93) | 1.48  (0.96 – 2.28) | 0.0765 |
| **E-cigarette Use** | **Past 12-month** | 3415 | 9.66  (9.26 - 10.06) | 32 | 4.98  (2.69 - 7.26) | 1.56  (0.89 – 2.73) | 0.1181 |
|  | *Current   combustible  smoking****^f^*** | 937 | 30.77  (28.70 - 32.83) | 26 | 80.74  (64.19 - 97.28) | – | – |
|  | *Former  combustible   smoking****^g^*** | 1027 | 35.74  (33.78 - 37.70) | 4 | 17.44  (0.91 - 33.98) | – | – |
| **Pouch Use** | **Past 12-month** | 439 | 1.35  (1.16 - 1.54) | 7 | 1.19  (0.09 - 2.28) | 2.62  (0.86 – 7.99) | 0.0897 |
|  | *Current   combustible  smoking****^f^*** | 123 | 29.60  (24.34 - 34.86) | 4 | 60.15  (11.72 - 100.00) | – | – |
|  | *Former  combustible   smoking****^g^*** | 152 | 42.62  (36.71 - 48.54) | 1 | 9.69  (0.00 - 31.63) | – | – |
| **Quit Attempts** | | | | | | | |
| **Combustible Quit Attempt^h^** | **Past 12-month** | 1805 | 31.76  (30.28 - 33.24) | 62 | 41.99  (32.26 - 51.71) | 1.45  (0.97 - 2.18) | 0.0684 |
|  | *Achieved*  *Abstinence****^i^*** | 581 | 31.16 (28.39 - 33.93) | 12 | 17.27  (5.42 - 29.13) | – | – |
| **Quit Strategies^j^** | | | | | | | |
| **Evidence-Based Strategy** | **Any Evidence-Based Strategy** | 481 | 28.10  (25.41 - 30.79) | 33 | 55.20  (39.42 - 70.99) | 2.35  (1.08 – 5.12) | 0.0320 |
|  | *Achieved*  *Abstinence****^k^*** | 130 | 27.77  (22.98 - 32.57) | 4 | 12.93  (0.00 - 27.08) | – | – |
|  | **Behavioral support** | 157 | 8.70  (7.06 - 10.34) | 14 | 21.62  (12.73 - 30.52) | 2.43 (1.19 – 4.93) | 0.0148 |
|  | *Achieved*  *Abstinence****^k^*** | 45 | 25.53  (16.86 - 34.21) | 2 | 15.15  (0.00 - 38.64) | – | – |
|  | **Mobile App** | 91 | 4.79  (3.57 - 6.00) | 9 | 14.96  (5.55 - 24.36) | 4.68  (1.83 – 11.99) | 0.0016 |
|  | *Achieved*  *Abstinence****^k^*** | 31 | 32.49  (18.98 - 46.02) | 2 | 21.91  (0.00 - 58.68) | – | – |
|  | **NRT**^l^ | 253 | 14.85  (12.78 – 16.93) | 20 | 34.20  (18.07 – 50.32) | 5.26  (0.28 – 97.15) | 0.2618 |
|  | *Achieved*  *Abstinence****^k^*** | 61 | 26.72  (20.59 – 32.85) | 1 | 8.34%  (0.00 – 25.45) | – | – |
|  | **Any Rx**^m^ | 115 | 19.87  (15.78 - 23.96) | 8 | 31.34  (6.49 - 56.18) | X | X |
|  | *Achieved*  *Abstinence****^k^*** | 26 | 10.57  (6.04 - 15.10) | 2 | 14.73  (0.00 - 35.89) | – | – |
| **Strategy: Other product use** | **Any Other Product** | 247 | 11.89  (10.28 - 13.50) | 5 | 9.86  (0.00 - 19.92) | 1.43  (0.35 – 5.89) | 0.6201 |
|  | *Achieved*  *Abstinence****^k^*** | 81 | 31.77  (25.58 - 37.97) | 0 |  |  |  |
|  | **E-cigarette** | 212 | 9.86  (8.41 - 11.31) | 4 | 7.71  (0.00 - 16.67) | 1.61  (0.34 – 7.71) | 0.5493 |
|  | *Achieved*  *Abstinence****^k^*** | 72 | 32.13  (25.14 - 39.12) | 0 | – | – | – |
|  | **Pouches** | 41 | 2.28  (1.40 - 3.15) | 1 | 2.16  (0.00 - 6.68) | 0.88  (0.05 – 14.44) | 0.9253 |
|  | *Achieved*  *Abstinence****^k^*** | 10 | 14.89  (7.04 - 22.73) | 0 | – | – | – |

**Note: X denotes models that failed due to convergence issues.** ^a^ Unweighted n. Ns and %s may not add up to full sample due to missing data. ^b^ Weighted column %. ^c^ Cancer type was only asked to respondents who endorsed a cancer diagnosis within the past 12 months (N=361). Tobacco-related cancers included bladder, cervix, colon, esophagus, kidney, larynx, liver, lung, mouth, pancreas, rectum, stomach, and throat. Non-tobacco related cancers included blood, bone, brain, breast, gallbladder, leukemia, lymphoma, melanoma, nervous system, ovarian, prostate, non-melanoma, unknown skin, soft tissue, testicular, thyroid, uterine, or other ^d^ Models included sex, age, race, ethnicity, education, and income, race, and ethnicity, and sex. ^e^ Cigar products include traditional cigars, cigarillos, and filtered cigars. ^f^ Current combustible smoking is defined as currently smoking cigarettes or cigar products everyday or some days (and for combustible cigarettes only, >100 lifetime cigarettes). Weighted %s indicate the proportion of those endorsing the primary non-cigarette product who endorse current combustible tobacco use. ^g^ Former combustible smoking is defined as having ever smoked cigarettes or cigar products fairly regularly (and for combustible cigarettes only, >100 lifetime cigarettes) but not within the past 12 months. Weighted %s indicate the proportion of those endorsing the primary non-cigarette product who endorse former combustible tobacco use. ^h^ Participants were asked about strategies used during their most recent combustible tobacco quit attempt. ^i^ Defined as those who endorsed past 12-month combustible tobacco use, but that they had quit at the time of the survey. Weighted %s indicate the proportion of column participants endorsing a quit attempt who had reported abstinence. ^j^ Quit strategies were only asked to participants who had endorsed a quit attempt in the past 12 months. Participants could indicate multiple strategies. ^k^ Weighted %s indicate the proportion of column participants who had endorsed this specific strategy and achieved abstinence. ^l^ Nicotine replacement therapy. ^m^ Chantix, varenicline, Wellbutrin, Zyban, and bupropion.

**Table 2b.** Tobacco use and quitting strategies of those diagnosed with cancer by cancer type: Results of weighted regression analyses

|  |  | **n^a^** | **Weighted % (95% CI)^b^** | **n^a^** | **Weighted %(95% CI)^b^** | **Adjusted OR (95% CI)^d^** | ***p*** |
| --- | --- | --- | --- | --- | --- | --- | --- |
| **Overall^c^** | N=361 | 268 | 80.26  (75.07 - 85.46) | 89 | 19.74  (14.54 - 24.93) |  |  |
| **Tobacco Use** | | | | | | | |
| **Cigarette Smoking** | **Past 12-month** | 71 | 11.69  (8.21 - 15.17) | 45 | 32.33  (19.37 - 45.28) | 2.46  (1.09 – 5.55) | 0.0303 |
| **Cigar Use^e^** | **Past 12-month** | 26 | 5.57  (3.04 - 8.09) | 12 | 6.87  (2.06 - 11.67) | 1.09  (0.42 – 2.87) | 0.8545 |
| **E-cigarette Use** | **Past 12-month** | 16 | 3.03  (1.06 - 4.99) | 15 | 12.15  (2.80 - 21.50) | X | X |
|  | *Current   combustible  smoking****^f^*** | 12 | 73.77  (45.45 -100.00) | 13 | 86.17  (64.16 - 100.00) | – | – |
|  | *Former  combustible   smoking****^g^*** | 2 | 22.46  (0.00 - 51.13) | 2 | 13.83  (0.00 - 35.84) | – | – |
| **Pouch Use** | **Past 12-month** | 3 | 0.78  (0.00 - 1.70) | 4 | 2.93  (0.00 - 6.26) | X | X |
|  | *Current   combustible  smoking****^f^*** | 2 | 81.82  (36.95 - 100.00) | 2 | 36.79  (0.00 - 92.13) | – | – |
|  | *Former  combustible   smoking****^g^*** | 0 | -- | 1 | 20.14  (0.00 - 61.81) | – | – |
| **Quit Attempts** | | | | | | | |
| **Combustible Quit Attempt^h^** | **Past 12-month** | 31 | 29.15  (17.21 - 41.10) | 30 | 59.63  (45.86 - 73.40) | X | X |
|  | *Achieved*  *Abstinence****^i^*** | 7 | 18.97  (3.10 -34.84) | 5 | 16.81  (0.00 - 34.42) | – | – |
| **Quit Strategies^j^** | | | | | | | |
| **Evidence- Based Strategy** | **Any Evidence-Based Strategy** | 15 | 2.35  (0.74 - 3.95) | 17 | 11.90  (5.95 - 17.84) | 2.25  (0.49 - 10.45) | 0.2901 |
|  | *Achieved*  *Abstinence****^k^*** | 2 | 7.88  (0.00 - 19.60) | 2 | 18.10  (0.00 - 40.88) | – | – |
|  | **Behavioral support** | 7 | 1.03  (0.07 - 1.99) | 7 | 4.62  (1.08 - 8.16) | X | X |
|  | *Achieved*  *Abstinence****^k^*** | 1 | 8.20  (0.00 - 25.21) | 1 | 21.45  (0.00 - 58.93) | – | – |
|  | **Mobile App** | 6 | 0.86  (0.00 - 1.74) | 3 | 2.58  (0.00 - 5.21) | X | X |
|  | *Achieved*  *Abstinence****^k^*** | 1 | 9.76  (0.00 - 30.65) | 1 | 38.47  (0.00 - 95.34) | – | – |
|  | **NRT**^l^ | 9 | 1.43  (0.24 - 2.62) | 10 | 7.08  (1.52 - 12.64) | X | X |
|  | *Achieved*  *Abstinence****^k^*** | 0 | – | 1 | 16.42  (0.00 - 47.97) | – | – |
|  | **Any Rx**^m^ | 8 | 1.22  (0.22 - 2.21) | 4 | 1.36  (0.00 - 3.11) | X | X |
|  | *Achieved*  *Abstinence****^k^*** | 2 | 28.10  (0.00 - 60.90) | 0 | – | – | – |
| **Strategy: Other product use** | **Any Other Product** | 1 | 0.21  (0.00 - 0.61) | 4 | 3.17  (0.00 - 7.30) | X | X |
|  | *Achieved*  *Abstinence****^k^*** | 0 | – | 0 | – | – | – |
|  | **E-cigarette** | 1 | 0.21  (0.00 - 0.61) | 3 | 2.30  (0.00 - 5.85) | X | X |
|  | *Achieved*  *Abstinence****^k^*** | 0 | – | 0 | – | – | – |
|  | **Pouches** | 0 | – | 1 | 0.88  (0.00 - 2.74) | X | X |
|  | *Achieved*  *Abstinence****^k^*** | 0 | – | 0 | – | – | – |

**Note: X denotes models that failed due to convergence issues.** ^a^ Unweighted n. Ns and %s may not add up to full sample due to missing data. ^b^ Weighted column %. ^c^ Cancer type was only asked to respondents who endorsed a cancer diagnosis within the past 12 months (N=361). Tobacco-related cancers included bladder, cervix, colon, esophagus, kidney, larynx, liver, lung, mouth, pancreas, rectum, stomach, and throat. Non-tobacco related cancers included blood, bone, brain, breast, gallbladder, leukemia, lymphoma, melanoma, nervous system, ovarian, prostate, non-melanoma, unknown skin, soft tissue, testicular, thyroid, uterine, or other ^d^ Models included sex, age, race, ethnicity, education, and income, race, and ethnicity, and sex. ^e^ Cigar products include traditional cigars, cigarillos, and filtered cigars. ^f^ Current combustible smoking is defined as currently smoking cigarettes or cigar products everyday or some days (and for combustible cigarettes only, >100 lifetime cigarettes). Weighted %s indicate the proportion of those endorsing the primary non-cigarette product who endorse current combustible tobacco use. ^g^ Former combustible smoking is defined as having ever smoked cigarettes or cigar products fairly regularly (and for combustible cigarettes only, >100 lifetime cigarettes) but not within the past 12 months. Weighted %s indicate the proportion of those endorsing the primary non-cigarette product who endorse former combustible tobacco use. ^h^ Participants were asked about strategies used during their most recent combustible tobacco quit attempt. ^i^ Defined as those who endorsed past 12-month combustible tobacco use, but that they had quit at the time of the survey. Weighted %s indicate the proportion of column participants endorsing a quit attempt who had reported abstinence. ^j^ Quit strategies were only asked to participants who had endorsed a quit attempt in the past 12 months. Participants could indicate multiple strategies. ^k^ Weighted %s indicate the proportion of column participants who had endorsed this specific strategy and achieved abstinence. ^l^ Nicotine replacement therapy. ^m^ Chantix, varenicline, Wellbutrin, Zyban, and bupropion.

**Survey Items**

**Note:** PATH questionnaires and data are publicly available, with further details provided (https://doi.org/10.3886/ICPSR36498.v22). For all below items, responses of “Don’t know” and “refused” were coded as missing in the present analyses.

1) Age
   a) Age for adults was derived from birthdate.
2) Sex
   a) Adults self-reported sex.

| What is your sex? | |
| --- | --- |
| 1 | Male |
| 2 | Female |
|  | DON'T KNOW |
|  | REFUSED |

3) Race
   a) Adults self-reported race.
   b) Responses 3-14 were coded as “Other” in the final public dataset.
   c) **Note:** Race and Ethnicity were included in the PATH study to ensure a representative sample, and to allow for analyses of differences in tobacco use and health outcomes (see Hyland et al., doi: 10.1136/tobaccocontrol-2016-052934).

| What is your race? Choose all that apply. | |
| --- | --- |
| 1 | White |
| 2 | Black or African American |
| 3 | American Indian or Alaska Native |
| 4 | Asian Indian |
| 5 | Chinese |
| 6 | Filipino |
| 7 | Japanese |
| 8 | Korean |
| 9 | Vietnamese |
| 10 | Other Asian |
| 11 | Native Hawaiian |
| 12 | Guamanian or Chamorro |
| 13 | Samoan |
| 14 | Other Pacific Islander |
|  | DON'T KNOW |
|  | REFUSED |

4) Ethnicity
   a) Adults self-reported ethnicity.
   b) Responses 2-5 were coded as “Hispanic” in the final public dataset.

| Are you Hispanic, [Latino/Latina/Latino or Latina], or of Spanish origin? Choose all that apply. | |
| --- | --- |
| 1 | No, not of Hispanic, [Latino/Latina/Latino or Latina], or Spanish origin |
| 2 | Yes, Mexican, Mexican American, [Chicano/Chicana/Chicano or Chicana] |
| 3 | Yes, Puerto Rican |
| 4 | Yes, Cuban |
| 5 | Yes, another Hispanic, [Latino/Latina/Latino or Latina], or Spanish origin |
|  | DON'T KNOW |
|  | REFUSED |

5) Education
   a) Adults self-reported education.
   b) In the present analyses, responses 1-2 were coded as “Less than high school (HS),” Reponses 3-4 were coded as “HS or GED,” responses 5-7 were coded as “Some college,” and responses 8-11 were coded as “College Grad.”

| What is the highest grade or level of school you have completed? | |
| --- | --- |
| 1 | Less than high school |
| 2 | Some high school, no diploma |
| 3 | GED |
| 4 | High school graduate-diploma |
| 5 | Some college but no degree |
| 6 | Associate degree-occupational/vocational |
| 7 | Associate degree-academic program |
| 8 | Bachelor's degree (ex: BA, AB, BS) |
| 9 | Master's degree (ex: MA, MS, MEng, MEd, MSW) |
| 10 | Professional school degree (ex: MD, DDS, DVM, JD) |
| 11 | Doctorate degree (ex: PhD, EdD) |
|  | DON'T KNOW |
|  | REFUSED |

6) Income
   a) Adults self-reported income.
   b) All participants were provided with this orientation text: *This is the total income before taxes of all persons in your household combined. Please include money from jobs, relatives, pensions, dividends, interest, social security payments or retirement benefits, net income from business, farm or rent, and any other money received by household members.*
   c) Responses 1-5 were coded as “<$50k” and responses 6-10 were coded as “$50k+.”

| Which of the following categories best describes your total **household** income in the past 12  months? | |
| --- | --- |
| 1 | Less than $10,000 |
| 2 | $10,000 to $14,999 |
| 3 | $15,000 to $24,999 |
| 4 | $25,000 to $34,999 |
| 5 | $35,000 to $49,999 |
| 6 | $50,000 to $74,999 |
| 7 | $75,000 to $99,999 |
| 8 | $100,000 to $149,999 |
| 9 | $150,000 to $199,999 |
| 10 | $200,000 or more |
|  | DON'T KNOW |
|  | REFUSED |

7) Past 12-month cancer diagnosis
   a) Adults self-reported whether or not they had seen a physician in the past 12 months.

| In the past 12 months, have you seen a medical doctor, nurse, or other health professional? | |
| --- | --- |
| 1 | Yes |
| 2 | No |
|  | DON'T KNOW |
|  | REFUSED |

b) Of those who had, participants self-reported cancer diagnosis

| In the past 12 months, have you been told by a doctor, nurse, or other health professional that you had cancer? | |
| --- | --- |
| 1 | Yes |
| 2 | No |
|  | DON'T KNOW |
|  | REFUSED |

c) Of those who had reported a cancer diagnosis, participants self-reported cancer site

| What kind of cancer were you told that you had? Choose all that apply | | Coded Cancer Type |
| --- | --- | --- |
| 1 | Bladder | Tobacco-related |
| 2 | Blood | Non-tobacco related |
| 3 | Bone | Non-tobacco related |
| 4 | Brain | Non-tobacco related |
| 5 | Breast | Non-tobacco related |
| 6 | Cervix (Cervical) | Tobacco-related |
| 7 | Colon | Tobacco-related |
| 8 | Esophagus (Esophageal) | Tobacco-related |
| 9 | Gallbladder | Non-tobacco related |
| 10 | Kidney | Tobacco-related |
| 11 | Larynx/Windpipe | Tobacco-related |
| 12 | Leukemia | Non-tobacco related |
| 13 | Liver | Tobacco-related |
| 14 | Lung | Tobacco-related |
| 15 | Lymphoma/Hodgkin’s disease | Non-tobacco related |
| 16 | Mouth/tongue/lip | Tobacco-related |
| 17 | Nervous system | Non-tobacco related |
| 18 | Ovary (ovarian) | Non-tobacco related |
| 19 | Pancreas (Pancreatic) | Tobacco-related |
| 20 | Prostate | Non-tobacco related |
| 21 | Rectum (Rectal) | Tobacco-related |
| 22 | Skin (Melanoma) | Non-tobacco related |
| 23 | Skin (Non-melanoma) | Non-tobacco related |
| 24 | Skin (Don’t know what kind) | Non-tobacco related |
| 25 | Soft tissue (muscle or fat) | Non-tobacco related |
| 26 | Stomach | Tobacco-related |
| 27 | Testis (Testicular) | Non-tobacco related |
| 28 | Throat | Tobacco-related |
| 29 | Thyroid | Non-tobacco related |
| 30 | Uterus (Uterine) | Non-tobacco related |
| 31 | Other | Non-tobacco related |
|  | DON'T KNOW |  |
|  | REFUSED |  |

8) Past 12-month tobacco product use
   a) Adults self-reported each product used in the past 12 months.
   b) Cigarettes
      i) All participants were provided with the below orientation text and a generic image:
         (1) *The next questions are about your experience with cigarettes.*

| In the past 12 months, have you smoked a cigarette, even one or two puffs? | |
| --- | --- |
| 1 | Yes |
| 2 | No |
|  | DON'T KNOW |
|  | REFUSED |

c) Cigars
   i) For the present analysis, affirmative responses to traditional cigar, cigarillo, and filtered cigar categories were coded as past 12-month cigar use.
   ii) All participants were provided with the below orientation text and generic images:
       (1) *The next questions are about traditional cigars, cigarillos, and filtered cigars. These products go by lots of different names, so please use these descriptions and photos to understand what they are.
           Traditional cigars contain tightly rolled tobacco that is wrapped in a tobacco leaf. Some common brands of cigars include Macanudo, Romeo y Julieta, Arturo Fuente, and Cohiba, but there are many others.*

| In the past 12 months, have you smoked a traditional cigar, even one or two puffs? | |
| --- | --- |
| 1 | Yes |
| 2 | No |
|  | DON'T KNOW |
|  | REFUSED |

(2) *Cigarillos and filtered cigars are smaller than traditional cigars. They are usually brown. Some are the same size as cigarettes, and some come with filters or with plastic or ooden tips. Some common brands are Black & Mild, Swisher Sweets, Dutch Masters, Phillies Blunts, Zig Zag, and Cheyenne.*

| In the past 12 months, have you smoked a cigarillo or filtered cigar, even one or two puffs? | |
| --- | --- |
| 1 | Yes |
| 2 | No |
|  | DON'T KNOW |
|  | REFUSED |

d) Oral nicotine pouches
   i) All participants were provided with the below orientation text and a generic image:
      (1) *The next questions ask about small, white pouches that contain nicotine which users place in their mouth.* ***Nicotine pouches*** *are different from other smokeless tobacco products such as snus, dip, or chew, because they do not contain any ground tobacco leaf. Common brands include Zyn, on!, or Velo, but there are many others.*

| In the past 12 months, have you used nicotine pouches, even one or two times? | |
| --- | --- |
| 1 | Yes |
| 2 | No |
|  | DON'T KNOW |
|  | REFUSED |

9) Combustible tobacco use status
   a) In the present analyses, current combustible use is defined as currently smoking cigarettes or cigar products (including traditional cigars, cigarillos, or filtered cigars) everyday or some days (and for combustible cigarettes only, >100 lifetime cigarettes)
   b) In the present analysis, former combustible use is defined as having ever smoked cigarettes or cigar products fairly regularly (and for combustible cigarettes only, >100 lifetime cigarettes) but not within the past 12 months.

| How many cigarettes have you smoked in your entire life? A pack usually has 20 cigarettes in it. | |
| --- | --- |
| 1 | 1 or more puffs but never a whole cigarette |
| 2 | 1 to 10 cigarettes (about ½ pack total) |
| 3 | 11 to 20 cigarettes (about ½ pack to 1 pack) |
| 4 | 21 to 50 cigarettes (more than 1 pack but less than 3 packs) |
| 5 | 51 to 99 cigarettes (more than 2 ½ packs but less than 5 packs) |
| 6 | 100 or more cigarettes (5 packs or more) |
|  | DON'T KNOW |
|  | REFUSED |

| Do you now smoke cigarettes...? | | | | |
| --- | --- | --- | --- | --- |
| 1 | | Every day | |  |
| 2 | Some days | |  |  |
| 3 | Not at all | |  |  |
|  | DON'T KNOW | |  |  |
|  | | REFUSED | |  |

| Do you now smoke traditional cigars/cigarillos/filtered cigars...? | | | | |
| --- | --- | --- | --- | --- |
| 1 | | Every day | |  |
| 2 | Some days | |  |  |
| 3 | Not at all | |  |  |
|  | DON'T KNOW | |  |  |
|  | | REFUSED | |  |

| Have you ever smoked cigarettes fairly regularly? | |
| --- | --- |
| 1 | Yes |
| 2 | No |
|  | DON'T KNOW |
|  | REFUSED |

| Have you ever smoked traditional cigars/cigarillos/filtered cigars fairly regularly? | |
| --- | --- |
| 1 | Yes |
| 2 | No |
|  | DON'T KNOW |
|  | REFUSED |

10) Abstinence Status
    a) Adults who reported past 12-month combustible tobacco (cigarettes, traditional cigars, cigarillos, and filtered cigars) were asked the following:

| Have you completely quit smoking cigarettes/traditional cigars/cigarillos/filtered cigars? | |
| --- | --- |
| 1 | Yes |
| 2 | No |
|  | DON'T KNOW |
|  | REFUSED |

11) Quit Attempt Status
    a) Adults who reported current combustible tobacco (cigarettes, traditional cigars, cigarillos, and filtered cigars) use were asked the following:

| In the past 12 months have you tried to quit smoking? | |
| --- | --- |
| 1 | Yes |
| 2 | No |
|  | DON'T KNOW |
|  | REFUSED |

b) Adults who reported combustible tobacco product use in the past 12-months, but had reported that they had quit at the time of the survey, were asked the below question. For the present analysis, responses >0 were coded as having made a quit attempt.

| In total, how many times in the past 12 months have you tried to quit cigarettes/traditional cigars/cigarillos/filtered cigars completely? | |
| --- | --- |
| ___________ | Up to 3 digits permitted |

12) Quit Methods
    a) Adults who reported a quit attempt in the past 12-months were provided with the below orientation text:
       i) *Thinking back to*
          (1) *the time you tried to quit (for those who reported 1 past-year quit attempt)*
          (2) *the last time you tried to quit (for those who reported >1 past-year quit attempt*
          (3) *when you quit (for those who reported that they quit)*
       ii) *the past 12 months, did you use any of the following to help you:*
    b) Participants responded to each of the following:

| counseling, a telephone help line or quit line, books, pamphlets, videos, a quit tobacco clinic, class, or support group, or an internet or web-based program? | |
| --- | --- |
| 1 | Yes |
| 2 | No |
|  | DON'T KNOW |
|  | REFUSED |

| an app on a tablet computer or smartphone to help you quit? | |
| --- | --- |
| 1 | Yes |
| 2 | No |
|  | DON'T KNOW |
|  | REFUSED |

c) Participants were provided with the following orientation text:
   i) *Thinking back to*
      (1) *the time you tried to quit (for those who reported 1 past-year quit attempt)*
      (2) *the last time you tried to quit (for those who reported >1 past-year quit attempt*
      (3) *when you quit (for those who reported that they quit)*
   ii) *the past 12 months, did you use:*
d) Participants who selected e-cigarettes (#3) or oral nicotine pouches (#12) below were coded as having used such products to quit combustible tobacco

| a different product [including e-cigarettes or other electronic nicotine products] to help you [stop/quit]? Choose all that apply. | |
| --- | --- |
| 1 | I did not use a different product to help me stop/quit |
| 2 | Cigarettes |
| 3 | E-cigarettes or other electronic nicotine products |
| 4 | Traditional cigars |
| 5 | Cigarillos |
| 6 | Filtered cigars |
| 7 | Pipe tobacco |
| 8 | Hookah |
| 9 | Snus |
| 10 | Smokeless tobacco (such as moist snuff, dip, spit, or chew) |
| 11 | IQOS |
| 12 | Nicotine pouches (such as Zyn, on!, or Velo) |
|  | DON'T KNOW |
|  | REFUSED |

e) Participants who reported any past 12-month use of nicotine replacement therapy or a prescription drug were provided with the following orientation text:
   i) *Thinking back to*
      (1) *the time you tried to quit (for those who reported 1 past-year quit attempt)*
      (2) *the last time you tried to quit (for those who reported >1 past-year quit attempt*
      (3) *when you quit (for those who reported that they quit)*
   ii) *the past 12 months:*

| did you use a nicotine patch, gum, inhaler, nasal spray or lozenge? | |
| --- | --- |
| 1 | Yes |
| 2 | No |
|  | DON'T KNOW |
|  | REFUSED |

| did you use Chantix, varenicline, Wellbutrin, Zyban, or bupropion? | |
| --- | --- |
| 1 | Yes |
| 2 | No |
|  | DON'T KNOW |
|  | REFUSED |
